# Supplementary material for: Integrative Analysis of Transcriptome and Metabolome Reveals Molecular Responses in Eriocheir sinensis with Hepatopancreatic Necrosis Disease
Source: Biology (Basel). 2022 Aug 26;11(9):1267. doi: 10.3390/biology11091267 (PMC9495758; doi:10.3390/biology11091267)
Supplement: Supplementary file 1 [file biology-11-01267-s001.zip › biology-1856183-supplementary/biology-1856183-supplementary-proofreading done/supplementary files/Fig.S1, Fig.S2.pdf]

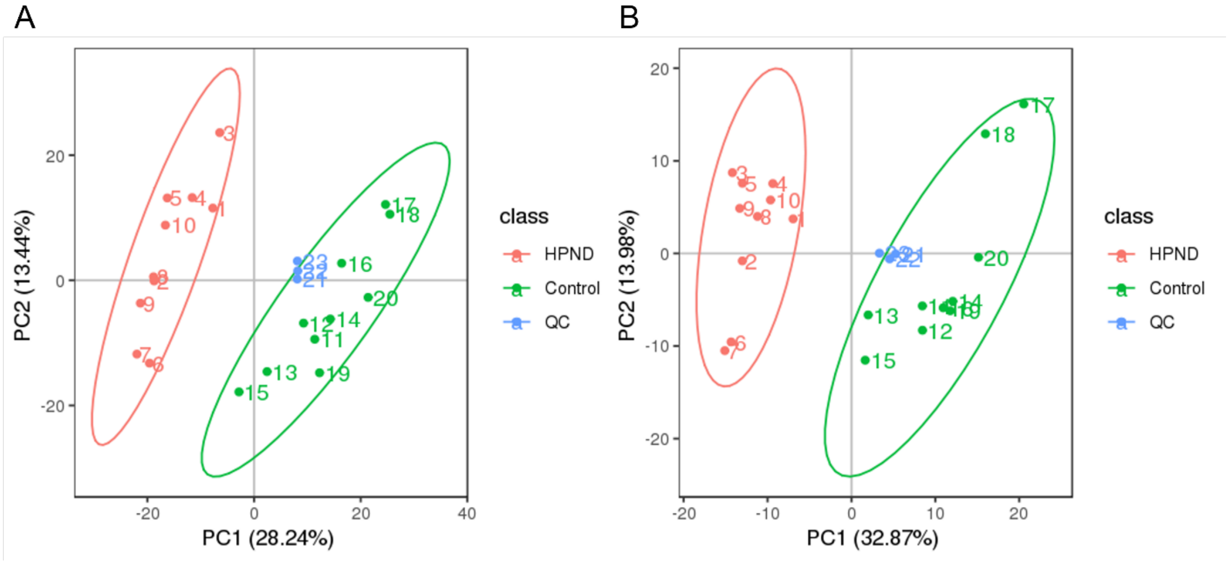

Figure S1. PCA score plots in different groups (A POS mode, B NEG mode).

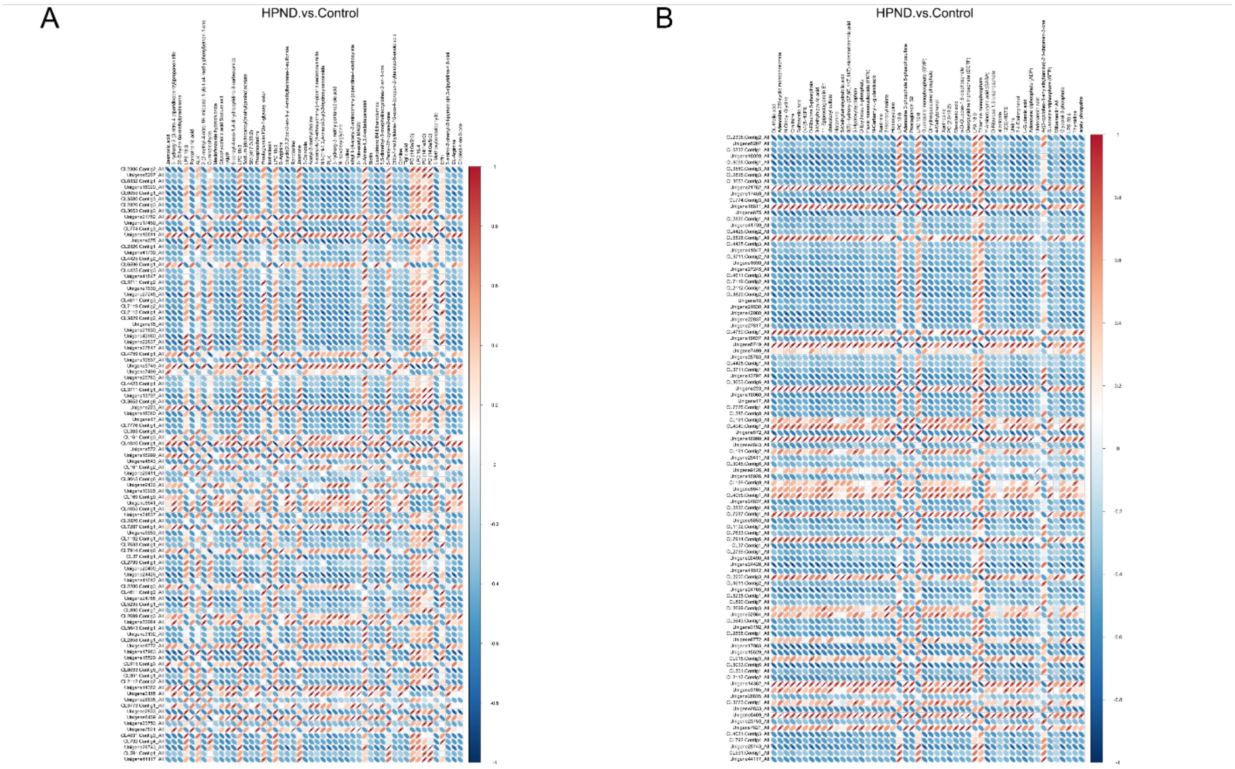

Figure S2. The heat plot of the correlations between the top 50 differentially expressed metabolites (rows) and top 100 differentially expressed genes (columns). The red and blue colors show the positively and negative correlation between transcriptomics and metabolomics data (A POS mode, B NEG mode).
