# Supplementary figures and images for: Integrative Analysis of Transcriptome and Metabolome Reveals Molecular Responses in Eriocheir sinensis with Hepatopancreatic Necrosis Disease
Source: Biology (Basel). 2022 Aug 26;11(9):1267. doi: 10.3390/biology11091267 (PMC9495758; doi:10.3390/biology11091267)

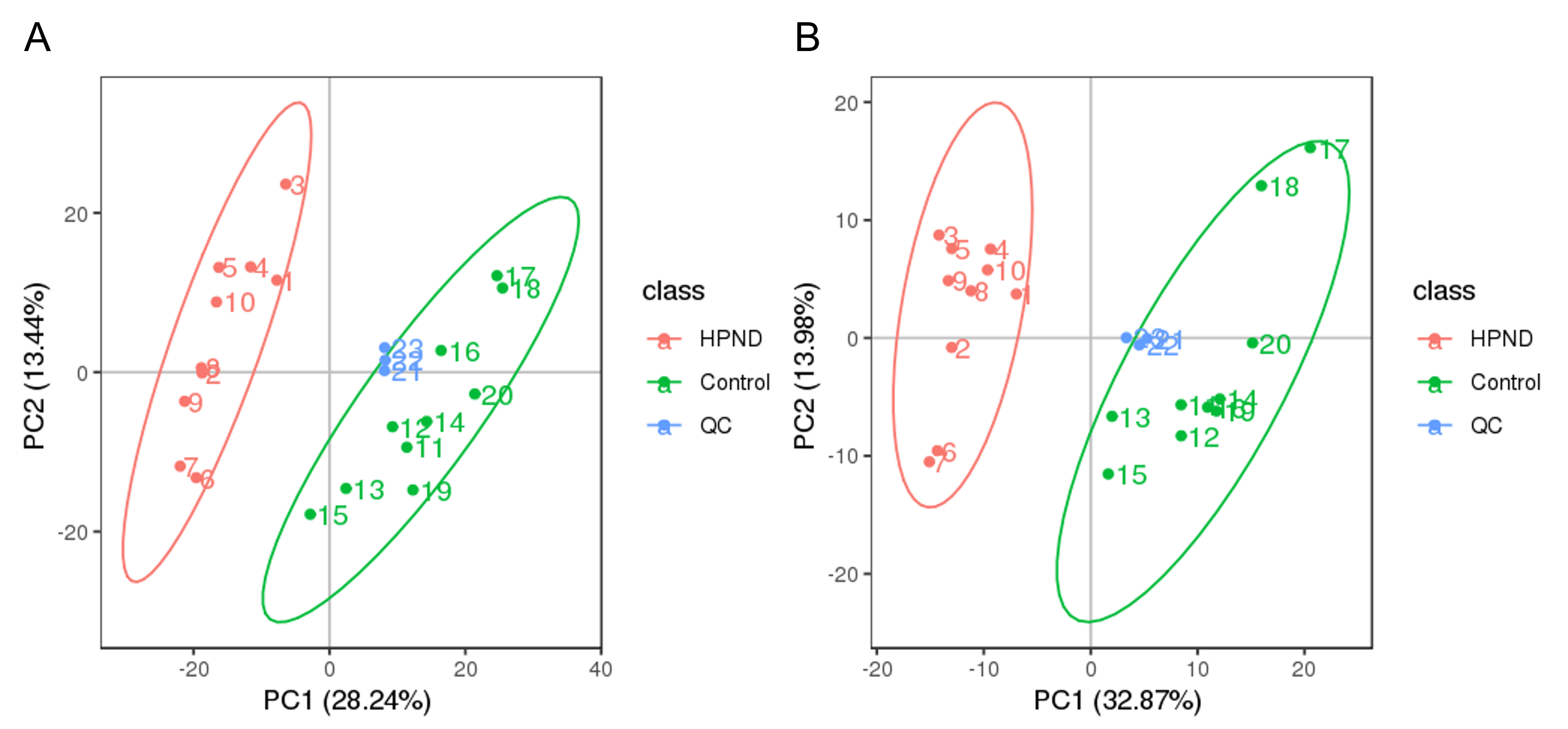

Supplement: Supplementary file 1 [file biology-11-01267-s001.zip › biology-1856183-supplementary/biology-1856183-supplementary-proofreading done/supplementary files/Fig.S1.png]

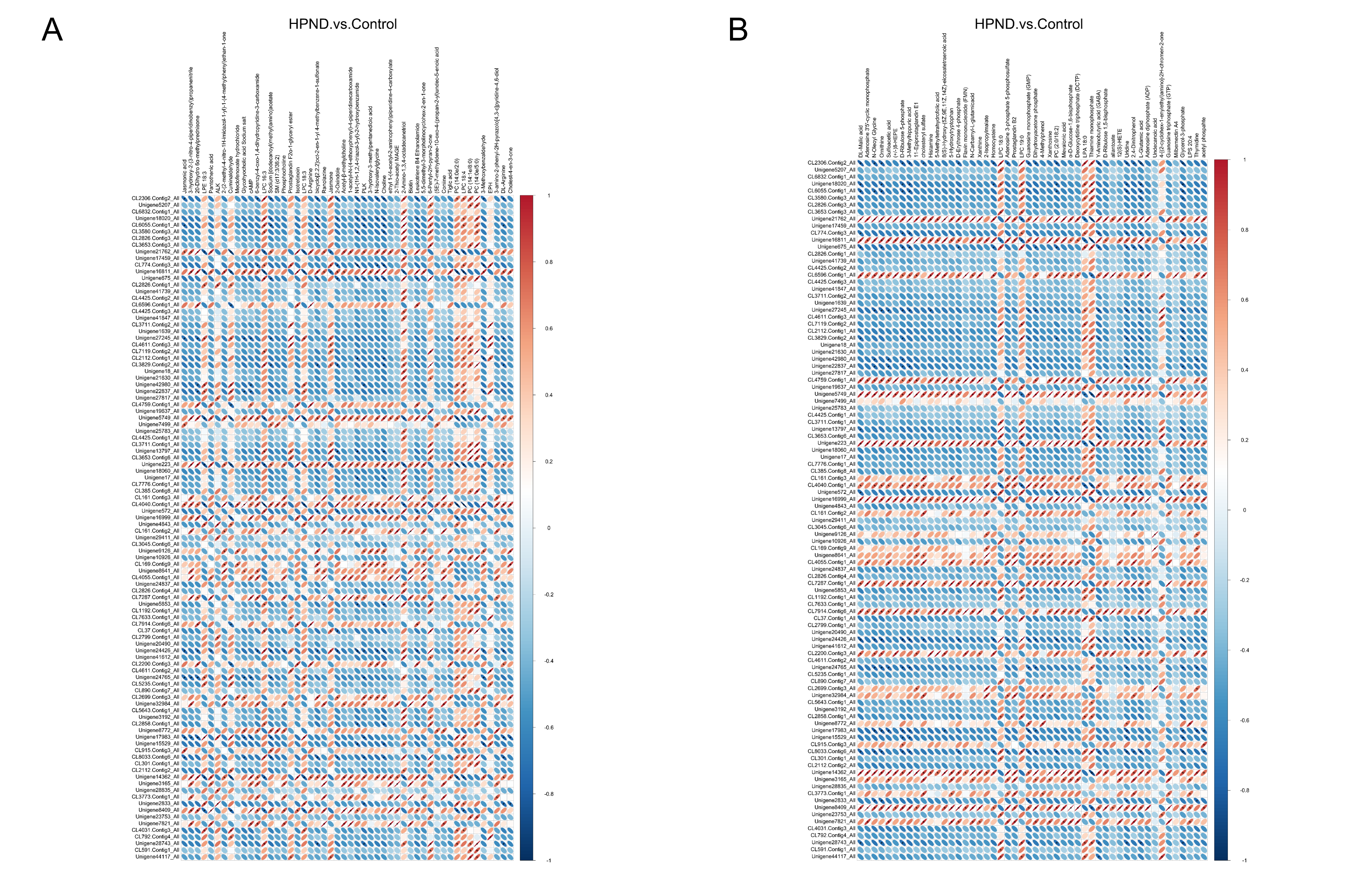

Supplement: Supplementary file 1 [file biology-11-01267-s001.zip › biology-1856183-supplementary/biology-1856183-supplementary-proofreading done/supplementary files/Fig.S2.png]
